# Zementembolie in die V. cava nach Pedikelschraubenaugmentation

**DOI:** 10.1007/s00113-020-00920-5

**Published:** 2020-11-17

**Authors:** S. David, F. X. Kleber

**Affiliations:** 1 Klinik für Unfallchirurgie und Orthopädie, Ev. Krankenhaus Paul-Gerhardt Stift, Paul-Gerhardt-Str. 42–45, 06886 Lutherstadt Wittenberg, Deutschland; 2 Klink für Innere Medizin III (Kardiologie), Ev. Krankenhaus Paul-Gerhardt Stift, Lutherstadt Wittenberg, Deutschland

**Keywords:** Wirbelkörperfraktur, Fixateur interne, Computertomografie, Endovaskuläre Entfernung, Vertebral body fracture, Fixater internal, Computed tomography, Endovascular removal

## Abstract

Eine 52-jährige Frau erlitt nach einem Sturz neben einer stabilen Fraktur von Brustwirbelkörper (BWK) 12 eine instabile Fraktur von Lendenwirbelkörper (LWK) 3 ohne neurologische Ausfälle. Neben der Ballonkyphoplastie von BWK 12 wurde die perkutane Fixateur-interne-Instrumentierung von LWK 2–4 mit zementaugmentierten Pedikelschrauben vorgenommen. Hierbei kam es zu Zementaustritten in die V. cava inferior. Diese Zementanteile wurden nach beginnender Ablösung endovaskulär entfernt. Der postinterventionelle Verlauf war unkompliziert.

## Abstract (en)

After a fall a 52-year-old female patient suffered an unstable fracture of lumbar vertebral body 3 and a stable fracture of thoracic vertebral body 12 without neurological deficits. In addition to the balloon kyphoplasty of thoracic vertebral body 12, percutaneous fixator internal instrumentation of lumbar vertebral bodies 2–4 was carried out with cement-augmented pedicle screws. Cement leakage into the inferior vena cava occurred. After the onset of detachment of the cement parts, we decided on an endovascular removal using the sling technique. The postinterventional course was uncomplicated.

## Anamnese

**Figure 1:**
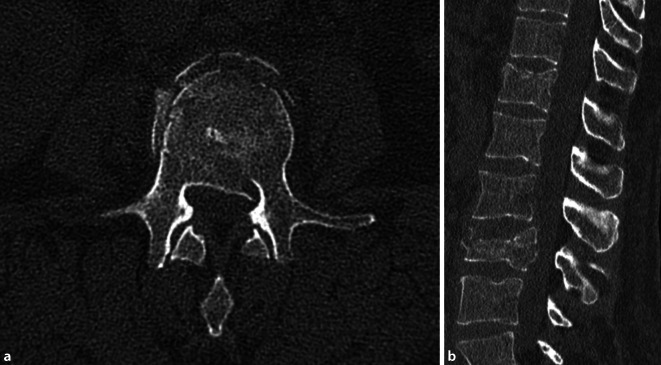

Es stellte sich eine 52-jährige Frau nach Treppensturz am Vortag wegen lumbaler Rückenschmerzen vor; neurologische Ausfälle bestanden nicht. Röntgen und Computertomografie (CT) zeigten eine stabile Fraktur des Wirbelkörpers BWK 12 (AOSpine A3: inkompletter Berstungsbruch, OF-Klassifikation [Arbeitsgruppe Osteoporotische Frakturen der Sektion Wirbelsäule der Deutschen Gesellschaft für Orthopädie und Unfallchirurgie]‑3) sowie eine instabile Fraktur des Wirbelkörpers LWK 3 (AOSpine A4: kompletter Berstungsbruch, OF-Klassifikation 4) ([5, 6]; Abb. 1).

Anamnestisch waren eine medikamentös behandelte Osteoporose bei langjährigem Nikotinabusus und ein nicht näher bekannter und behandelter Herzscheidewanddefekt als Kleinkind bekannt.

## Therapie und Verlauf

**Figure 2:**
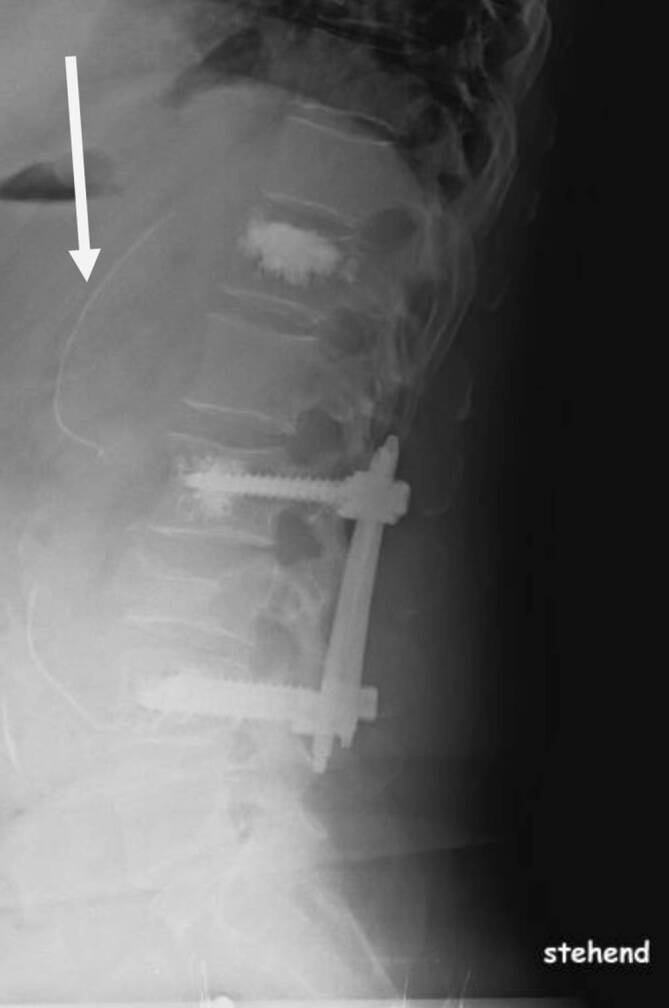

Wir empfahlen die Kyphoplastie von BWK 12 und Stabilisierung von LWK 3 durch einen Fixateur interne von LWK 2 auf LWK 4. Die operative Versorgung erfolgte am Aufnahmetag. Nach unkomplizierter Kyphoplastie von BWK 12 wurden die geschlossene Reposition und perkutane Fixateur-interne-Stabilisierung von LWK 3 mit Augmentation der 4 kanülierten/perforierten Pedikelschrauben (CD Horizon Longitude; Fa. Medtronic, Dublin, Irland) mit jeweils 1,5 ml niedrigviskösem Zement vorgenommen. Vor dem Einpressen des Zementes über die „bone filler“ wurde, wie empfohlen, 6 min bis zur zähflüssigen Konsistenz gewartet. Dies erfolgte unter gepulsterter Bildwandlerkontrolle, je Schraube wurden 1,5 ml Zement (Osteopal V, Fa. Heraeus, Hanau, Deutschland) genutzt; die Zementverteilung zeigte sich an gewünschter Stelle. Erst nach Beendigung der Zementierung fiel ein Austritt von 2 Zementfahnen nach ventral-kranial durch die Pedikelschraubenspitzen in LWK 2 und 4 offenbar über venöse Verbindungen in die V. cava auf. Nach abschließender Röntgendokumentation wurden die Weichteile verschlossen und die Narkose ausgeleitet. Neurologische Ausfälle oder klinische Hinweise für eine Lungenembolie bestanden postoperativ nicht (Abb. 2).

**Figure 3:**
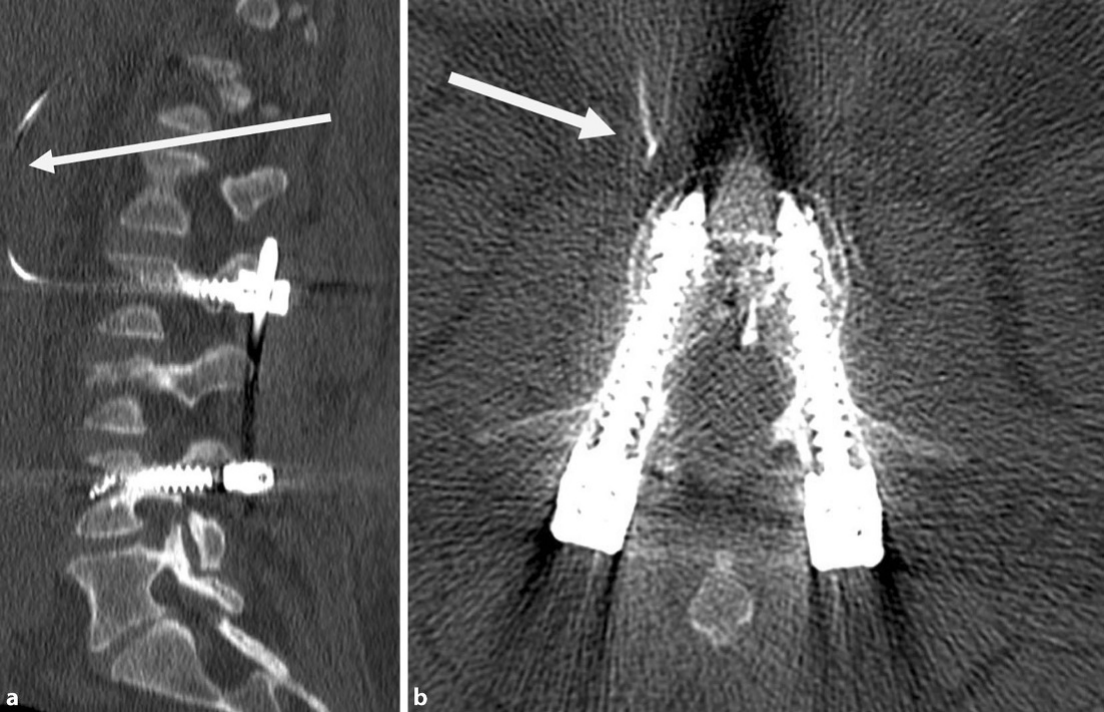

Das postoperative CT der LWS bestätigte die vermutete Zementembolie. Diese ging von den rechtsseitigen Pedikelschrauben in LWK 2 und 4 aus und erfolgte über paravertebrale Venen in die V. cava inferior. Die Schraubenlage und Frakturreposition waren regelrecht. Die Zementfahnen hatten Verbindung zu den Wirbelkörpern (Abb. 3). Eine klinische Symptomatik bezüglich einer Zementlungenembolie bestand nicht. Es erfolgte eine Thromboseprophylaxe mit Dalteparin 5000 I.E. einmal täglich.

Nach Diskussion des Falls in der Gefäßkonferenz wurde in Abwägung der möglichen Risiken und Notwendigkeit einer Zemententfernung (Wird dadurch eine Embolie provoziert? Wäre eine Zementlungenembolie überhaut symptomatisch? Kann alles Material überhaupt entfernt werden, und wenn ja, wie?) ein Eingriff zur Zemententfernung auch aufgrund der völligen Beschwerdefreiheit der Patientin zurückgestellt. Eine CT-Untersuchung von LWS, Thorax und Abdomen zur Verlaufskontrolle und zum Ausschluss einer Zementembolie wurde empfohlen.

**Figure 4:**
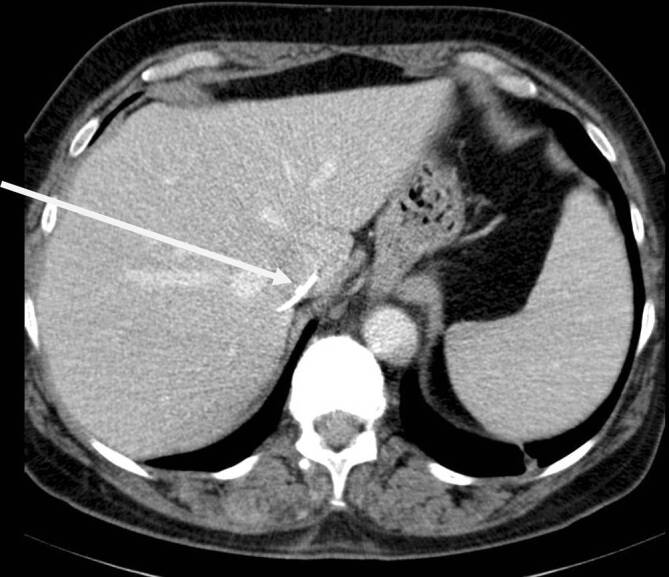

Dieses CT mit Kontrastmittel 5 Wochen nach dem Eingriff zeigte nun teilweise eine Verlagerung der Zementstränge nach zentral. Es bestand z. T. eine Verhakung der Zementspitzen in der Gefäßwand; weiter fanden sich kleine Zementanteile in der Lunge (Abb. 4).

Zur Prophylaxe einer weiteren Embolie, Gefäß- oder Herzverletzung durch größere Zementanteile wurde der Patientin nun die endovaskuläre Entfernung der kavalen Zementreste empfohlen.

**Figure 5:**
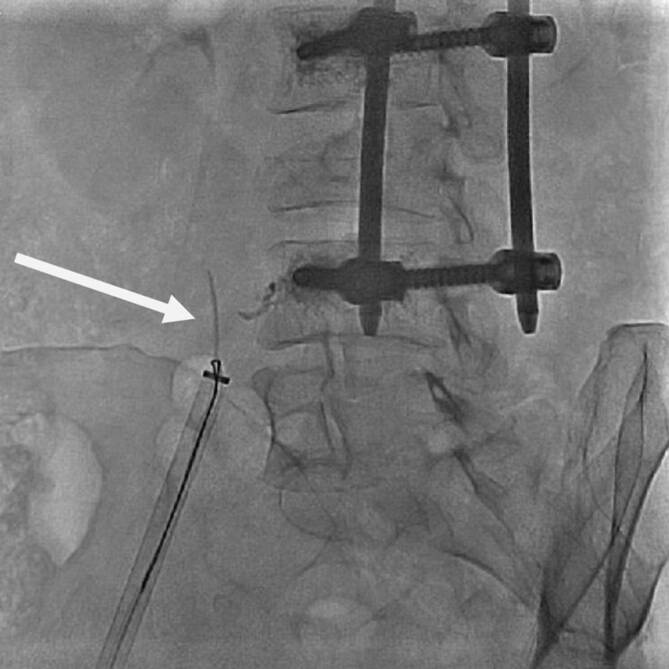

Dieser Eingriff erfolgte 2 Monate nach operativem Eingriff im Hybrid-OP in Analgosedierung und unter Narkosebereitschaft (Abb. 5).

**Figure 6:**
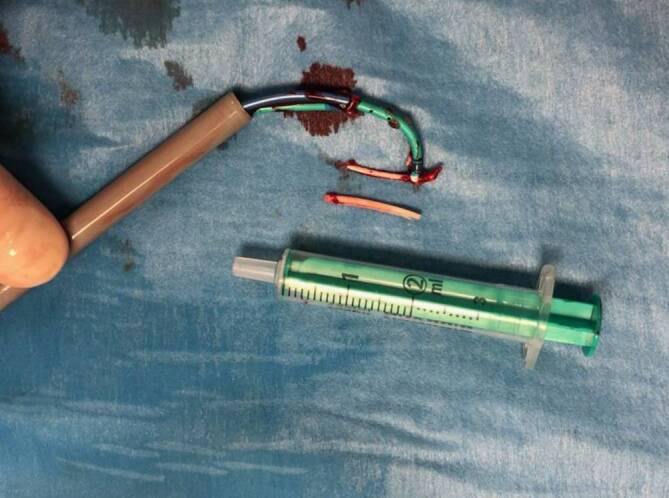

Es gelang im Rahmen des kathetergestützten Eingriffes die Entfernung des größten Zementrestes in Doppelschlingentechnik über eine Schleuse (Introducer Sheath with hydrophilic Coating, 18 F 28 cm; Fa. Medtronic, USA) im Bereich der rechten V. femoralis. Zum Schutz vor einer zentralen Embolisation wurde zunächst „off label“ ein Amplatzer cribriform ASD Occluder (Fa. Abbott Cardiovascular, Abbott Park, USA, normalerweise zum Verschluss eines persistierenden Foramen ovale genutzt) verwendet, da ein temporärer Kavaschirm als zu durchlässig eingeschätzt wurde und die Bergung des sehr rigiden Materials darin problematisch sein könnte. Der Amplatzer-Schirm wurde proximal der Läsionen platziert, entfaltet und dann an die Läsionen herangezogen. Er erwies sich aber als zu nachgiebig und weich, sodass mit 2 „snares“ (Schlingenkatheter, Amplatz Goose Neck Snare Kit 36 mm (120 cm) bzw. 25 mm (120 cm); Fa. Medtronic, Dublin, Irland) das größte der Zementstücke gefasst wurde. Eine Snare diente zum Halten des Fremdkörpers, die zweite zum Abbrechen bzw. Loslösen aus der Gefäßwand. Es gelang, das Zementstück mit beiden Snares in die Schleuse zurückzuziehen und mit der Schleuse zu entfernen (Abb. 6). Nach Hautverschluss mit einer Z‑Naht wurde ein Druckverband angelegt.

Eine Entfernung der in die Lunge embolisierten Zementanteile gelang nicht.

Der postoperative Verlauf war unauffällig. Neurologische Ausfälle oder klinische Zeichen einer Lungenembolie bestanden unverändert nicht. Die Patientin war unter milder Schmerzmedikation voll belastbar.

## Diskussion

Frakturen der Brust- und Lendenwirbelsäule können funktionell-konservativ behandelt werden. Instabile Wirbelkörperfrakturen werden operativ stabilisiert, bei neurologischem Defizit die entsprechenden Strukturen entlastet [6, 7]. Vorteile der operativen Therapie sind u. a. die Prophylaxe einer sekundären Frakturdislokation und Prävention neurologischer Defizite [6]. Minimal-invasive Verfahren haben eine hohe Bedeutung. Für osteoporotische Wirbelkörperfrakturen wird die Vertebroplastie und Kyphoplastie genutzt [6]. Bei instabiler Fraktur erfolgt zunächst die Stabilisierung durch den Fixateur interne. Bei Osteoporose kann die Stabilität durch Zementaugmentation der Pedikelschrauben erhöht werden [8].

Wir entschlossen uns bei instabiler Fraktur des Wirbelkörpers LWK 3 zu einer minimal-invasiven Fixateur-interne-Stabilisierung mit Zementaugmentation, dabei kam es zu Zementaustritten über die Schraubenspitzen in die V. cava inferior.

Zementaustritte im Rahmen der Augmentationen von Wirbelkörpern (Vertebroplastie, Kyphoplastie, Schraubenaugmentation) sind eine schwerwiegende Komplikation; laut Literatur werden sie in bis zu 11 % der Fälle beschrieben [1, 3]. Nur selten ist mit gravierenden Folgen (neurologisches Defizit, Embolie, Gefäßverletzung, Herzverletzung) zu rechnen, und nur 0,2 % der Zementaustritte werden klinisch symptomatisch (in [3, 4]).

Der venöse Zugang über die Leiste hat eine sehr geringe Komplikationsrate. Dieser Zugangsweg wird routinemäßig für eine Reihe von Eingriffen in der interventionellen Kardiologie bei strukturellen Herzkrankheiten wie bei Atrium-Septum-Defekt (ASD) und persistierendem Foramen ovale (PFO),  zum Verschluß des linken Vorhofohres (LAA) oder einem Mitral-Clipping u.v.a.m. genutzt, bei denen Komplikationen im Venensystem keine große Rolle spielen.

Blutungen an der Punktionsstelle oder lokale und retroperitoneale Hämatome sowie Lungenembolien durch bzw. nach einem Eingriff über die V. femoralis/V. cava sind äußerst selten und werden zusammen mit 0,6 % bei vergleichbaren Eingriffen genannt [6].

Um Zementaustritten bei Pedikelschraubenaugmentationen vorzubeugen, wird neben reduzierten Zementmengen (maximal 1–1,5 ml) und dem Abwarten einer ausreichenden Zementviskosität empfohlen, die Schrauben in unterschiedlichen Wirbelkörpern zu zementieren, um die Zementverteilung der einzelnen Schraube unter Bildwandler besser beurteilen und intravertebrale Druckspitzen vermeiden zu können [7]. In unserem Fall kam es trotz vorsichtigen Vorgehens intraoperativ zur Zementembolie, die erst zum Operationsende erkannt wurde.

Zur Prophylaxe einer Embolie größerer Zementreste in das Herz oder die Lunge entschieden wir uns zur – in unseren Augen eleganten – Entfernung der größten Zementreste durch ein minimal-invasives endovaskuläres Verfahren; ein ähnliches Vorgehen beschreiben Baumann und Kim [1, 2].

## Fazit

Bei einer Zementaugmentation von Fixateur-interne-Pedikelschrauben ist die Gefahr des Austritts in die paravertebralen Venenplexus und nachfolgend die V. cava mit drohender Embolie zu beachten. Eine postoperative CT-Untersuchung der in die Instrumentierung einbezogenen Wirbelkörper sollte immer erfolgen; bei einem Verdacht auf einen Zementaustritt ist ein CT von Thorax und Abdomen mit Kontrastmittel zur Diagnostik zu empfehlen. Unter Abwägung der Vor- und Nachteile ist zur Entfernung intravasaler Zementreste ein endovaskuläres Vorgehen eine Behandlungsoption.
